# Low-dose photon irradiation induces invasiveness through the SDF-1α/CXCR4 pathway in malignant mesothelioma cells

**DOI:** 10.18632/oncotarget.19134

**Published:** 2017-07-10

**Authors:** Yoshikane Yamauchi, Seyer Safi, Lena Orschiedt, Adriane Gardyan, Stephan Brons, Juliane Rieber, Nils H. Nicolay, Peter E. Huber, Martin Eichhorn, Hendrik Dienemann, Felix J.F. Herth, Klaus-Josef Weber, Jürgen Debus, Hans Hoffmann, Stefan Rieken

**Affiliations:** ^1^ Department of Thoracic Surgery, Thorax Clinic, Heidelberg University, Heidelberg, Germany; ^2^ Department of Radiation Oncology, University Hospital of Heidelberg, Heidelberg, Germany; ^3^ Heidelberg Ion Treatment Facility (HIT), Heidelberg, Germany; ^4^ Heidelberg Institute of Radiation Oncology (HIRO), Heidelberg, Germany; ^5^ Department of Molecular and Radiation Oncology, German Cancer Research Center (dkfz), Heidelberg, Germany; ^6^ Pneumology and Critical Care Medicine, Thorax Clinic, Heidelberg University, Heidelberg, Germany; ^7^ Translational Lung Research Center Heidelberg (TLRCH), Heidelberg, Germany, Member of the German Center for Lung Research (DZL)

**Keywords:** mesothelioma, photon irradiation, carbon ion irradiation, CXCR4, SDF-1α

## Abstract

**Background:**

Low-dose photon irradiation has repeatedly been suspected to increase a risk of promoting local recurrence of disease or even systemic dissemination. The purpose of this study was to investigate the motility of malignant pleural mesothelioma (MPM) cell lines after low-doses of photon irradiation and to elucidate the mechanism of the detected phenotype.

**Methods:**

H28 and H226 MPM cells were examined in clonogenic survival experiments and migration assays with and without various doses of photon and carbon ion irradiation. C-X-C chemokine receptor type 4 (CXCR4), SDF-1α, β_1_ integrin, α_3_ integrin, and α_5_ integrin expressions were analyzed by quantitative FACS analysis, ELISA and western blots. Apoptosis was assessed via Annexin-V-staining.

**Results:**

The migration of MPM cells was stimulated by both fetal bovine serum and by stromal cell-derived factor 1α (SDF-1α). Low doses of photon irradiation (1 Gy and 2 Gy) suppressed clonogenicity, but promoted migration of both H28 and H226 cells through the SDF-1α/CXCR4 pathway. Hypermigration was inhibited by the administration of CXCR4 antagonist, AMD3100. In contrast, corresponding doses of carbon ion irradiation (0.3 Gy and 1 Gy) suppressed clonogenicity, but did not promote MPM cell migration.

**Conclusion:**

Our findings suggest that the co-administration of photon irradiation and the CXCR4-antagonist AMD3100 or the use of carbon ions instead of photons may be possible solutions to reduce the risk of locoregional tumor recurrence after radiotherapy for MPM.

## INTRODUCTION

Malignant pleural mesothelioma (MPM) is a rare but aggressive neoplasm that has been the subject of considerable attention given its strong relationship to asbestos and its dismal outcome despite continuous efforts to intensify its treatment. Chemotherapy, radiotherapy, and surgery have been proven ineffective as single treatment modalities, but combined modality regimes have yielded minor improvement in disease progression [1]. However, no definite treatment guidelines have been established, because of the poor quality of the published evidence [2]. In particular, the impact of adjuvant high-dose hemithoracic radiotherapy after surgical complete resection following initial neoadjuvant chemotherapy remains controversial [3–6]. In addition, a recent phase 2 trial of postoperative radiotherapy in MPM showed no survival benefit [7]. It is also reported that, despite some small improvements in local control of patients with postoperative radiation, regional relapse remains the predominant pattern of tumor recurrence after multimodality treatment [8].

Photon beams are routinely used for external-beam radiotherapy. In order to predict and avoid radiation injury to healthy tissues, dose limitations for such tissues and organs have been described. During the past decades, innovative delivery techniques for photon irradiation, which is the most often used modality for radiation treatments, have evolved that allow dose accumulation within precisely contoured targets while uninvolved structures in close vicinity can be spared. Three-dimensional treatment planning, stereotactic setups and intensity-modulated radiotherapy (IMRT) have substantially improved dose deposition with minimized exposure of adjacent organs. However, some recent reports have still shown high rates of local in-field or marginal recurrences and significant toxicity after adjuvant radiotherapy using photon IMRT (16-48%) [9–11]. As the principal and unalterable physical characteristics of photons imply gradual energy deposition, a new modality – particle irradiation – has been suggested to continuously replace photon treatments: the physical and biological properties of particle irradiation facilitate dose-escalated treatments with significantly reduced dose exposure to uninvolved peritumoral tissues and organs.

The repeatedly reported notion of post-radiation tumor recurrences occurring mostly within areas of prior photon dose exposure has raised the question of whether mesothelioma cells might increase their motility and migrate from radiation fields before lethal doses have been administered during photon radiotherapy. Several authors have demonstrated in other malignancies, such as lung cancer [12–14], prostate cancer [15], melanoma [16], fibrosarcomas [17], and glioma [18], that low doses of photon irradiation can stimulate mechanisms of cellular migration and alter the peritumoral stroma into a promigratory milieu through integrin induction [12, 17], transformation of extracellular matrix proteins [13], activation of matrix-metalloproteinases [15, 16, 18], and stimulation of chemokine secretion. Among the many different chemokines, SDF-1α has repeatedly been identified to account for radiation-induced motility in thoracic malignancies [14].

Therefore, we hypothesized that low doses of photon irradiation might promote the migration of MPM cells. The purpose of this study was to investigate the motility of MPM cells after low doses of photon and particle irradiation and to elucidate the mechanisms of the detected phenotypes.

## RESULTS

### Mesothelioma cell migration was promoted by serum components

**Figure 1:**
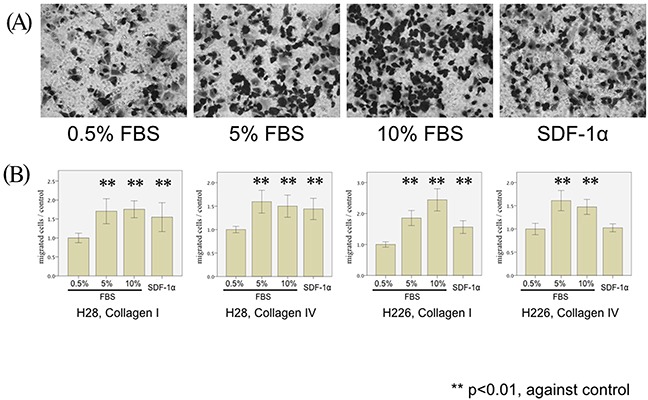
MPM cell migration stimulated by FBS and SDF-1α **(A)** H226 cells are microscopically analyzed after the 5-hour migration assay using 0.5% FBS (left), 5% FBS (center left), 10% FBS (center right), and SDF-1α (0.1μg/ml) (right) as chemoattractants and Collagen I-coated membranes (magnification x20). **(B)** H28 cell migration through Collagen I- (left Figure) and Collagen IV- (middle left figure) coated membranes and H226 cell migration through Collagen I- (middle right Figure) and Collagen IV- (right Figure) coated membranes were examined in the modified Boyden chamber experiments. The number of stimulated MPM cells (H28 and H226) transmigrated through collagen I- and collagen IV-coated membranes was counted. In each Figure, the average cell counts after the 5-hour migration assay using 0.5% FBS is defined as a control, and the normalized ratio of each cell count to the control is calculated.

In order to analyze the migration ability of mesothelioma, modified Boyden chamber assays were performed to analyze transmigration of H28 and H226 mesothelioma cells through 8-μm pore size polycarbonate membranes coated with collagen I and IV. Both cell lines were attracted by serum in a concentration-dependent manner (5% and 10%). Moreover, SDF-1α, which is a typical pro-migratory serum component, also promoted the migration of H28 through collagen I- and IV-coated membranes (p=0.007 and p<0.001, respectively) and H226 cells through collagen I-coated membranes (p<0.001) (Figure 1).

### Low-doses of photon irradiation enhance mesothelioma cell migration

**Figure 2:**
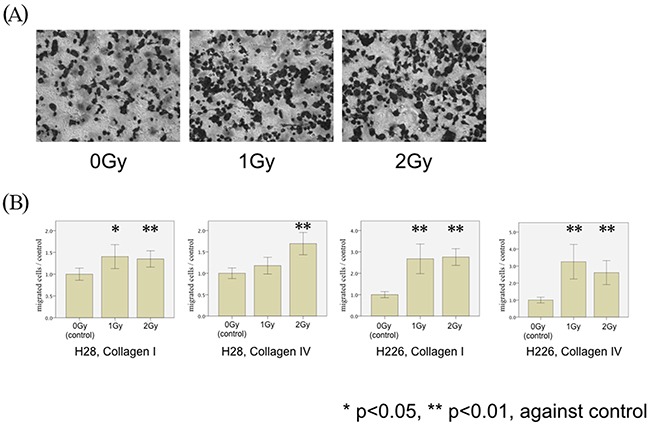
MPM cell migration following photon irradiation **(A)** H226 cells are microscopically analyzed without (left) and with irradiation by single doses of 1 Gy (middle) and 2 Gy (right) in modified Boyden chamber experiments using Collagen I-coated membranes (magnification x20). **(B)** H28 cell migration through Collagen I- (left Figure) and Collagen IV- (middle left Figure) coated membranes and H226 cells through Collagen I- (middle right Figure) and Collagen IV- (right Figure) coated membranes were examined in the modified Boyden chamber experiments. The number of irradiated MPM cells (H28 and H226) transmigrated through collagen I- and collagen IV-coated membranes was counted. The average number of migrated cells in the non-irradiated condition is defined as a control, and the normalized ratio of each cell count to the control is calculated.

We investigated SDF-1α-induced transmigration of both mesothelioma cell lines through membranes coated with collagen I and IV following single doses of photon irradiation (1 Gy and 2 Gy): Irradiation of both H28 and H226 cells with single photon doses of 1 Gy and 2 Gy increased transmigration through collagen I- and collagen IV-coated filters. Compared with non-irradiated cells, a significant increase of cell migration through collagen I-coated filters was noted after 1 Gy and 2 Gy in H28 cells (p=0.009 and p=0.003, respectively), and on collagen IV-coated filters after 2 Gy (p<0.001). In H226 cells, irradiation with 1 Gy and 2 Gy increased transmigration through both collagen I and IV-coated filters (p<0.001 and p=0.003, respectively) (Figure 2).

### Photon irradiation increased the expression of CXCR4, but not of α_3_, α_5_, or β_1_ integrins

**Figure 3:**
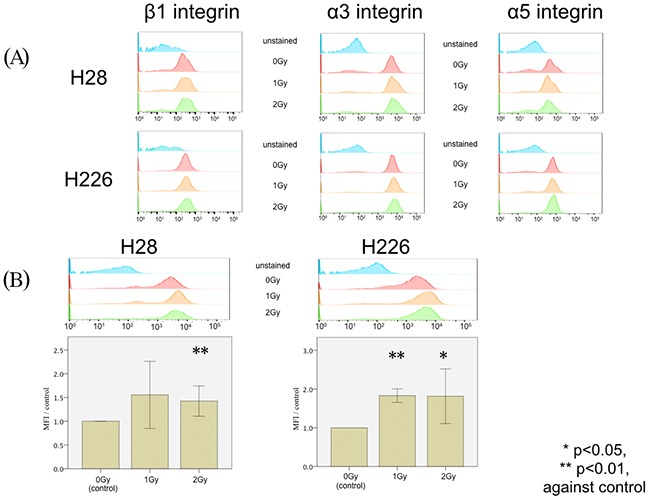
FACS analysis of photon-induced stimulation of expression in cell surface markers **(A)** FACS analysis of the expression of β1 integrin (left column), α3 integrin (middle column), and α5 integrin (right column) does not reveal any difference among H28 (upper row) or H226 cells (lower row) without irradiation, with 1 Gy irradiation, and with 2 Gy irradiation. Unstained cells were analyzed in the same experiment as a negative control. **(B)** FACS histogram analysis of CXCR4 expression (upper row) upon a single dose of 1 Gy irradiation and 2 Gy irradiation in H28 cells (left) and H226 cells (right) compared to non-radiated cells. Data are given as the normalized ratio of CXCR4 expression compared to non-irradiated cells.

In order to clarify the underlying mechanism of photon-induced hypermigration, FACS analyses were performed to investigate alterations in integrin expression caused by irradiation. No significant modifications of α_3_, α_5_, or β_1_ integrin expression were observed in both H28 and H226 cells following photon irradiation (Figure 3A). On the other hand, the expression of CXCR4 was significantly increased on H28 cells after single doses of 2 Gy photon irradiation (p=0.005) and on H226 cells after single doses of 1 Gy and 2 Gy photon irradiation (p<0.001 and p=0.033, respectively) (Figure 3A). Therefore, CXCR4 was selected for subsequent investigations.

### Inhibition of CXCR4 significantly impaired photon-induced hypermigration

**Figure 4:**
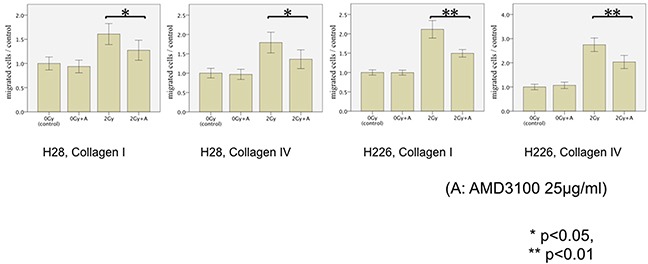
MPM cell migration following photon irradiation and treatment of cells with AMD3100 (25μg/ml) The transmigration of H28 cells through Collagen I-coated membrane (left), H28 cells through Collagen IV-coated membrane (middle left), H226 cells through Collagen I-coated membrane (middle right), and H226 cells through Collagen IV-coated membrane (right) is examined in the modified Boyden chamber experiments In each Figure, the average cell count of non-irradiated cells is defined as a control. Results are displayed as normalized ratio of cell counts compared to the control.

In order to inhibit CXCR4, we selected AMD3100 in this study. The exposure of both cell lines to AMD3100 24 hours before the migration assays almost fully reversed the detected phenotype of photon-induced migration and significantly inhibited the previously radiation-enhanced migration through collagen I/IV-coated membranes of both H28 and H226 cells after exposition to photon radiation with 2 Gy (H28: p=0.024 and p=0.016; H226: p<0.001 and p=0.001, respectively). However, AMD3100 did not affect the migration of non-irradiated MPM cells (Figure 4).

### Carbon-ion irradiation did not stimulate CXCR4-SDF1α signaling

**Figure 5:**
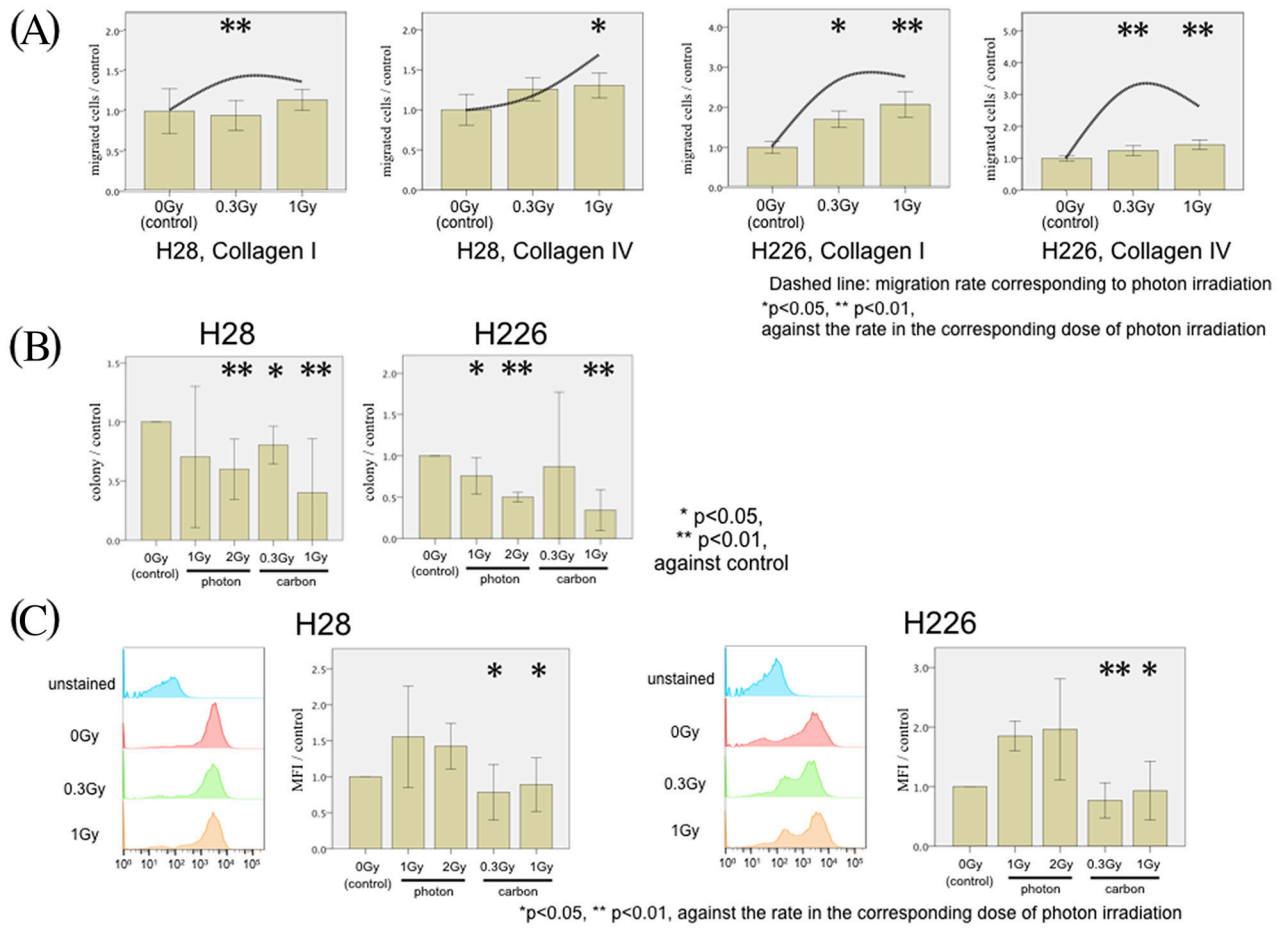
MPM cell migration following carbon irradiation **(A)** H28 cell migration through Collagen I- (left figure) and Collagen IV- (middle left figure) coated membranes and H226 cells through Collagen I- (middle right figure) and Collagen IV- (right figure) coated membranes are examined in the modified Boyden chamber experiments. Transmigrated MPM cells without irradiation (left bar) with 0.3 Gy carbon ion irradiation (middle bar) and with 1 Gy carbon ion irradiation (right bar) were counted. In each Figure, the average of the cell count without irradiation is defined as the control, and the normalized ratio of each cell count to the control is analyzed. The mean migration rate of corresponding photon irradiation is presented as a dashed line. **(B)** Analysis of clonogenic survival with and without photon and carbon ion irradiation in H28 and H226 cells. **(C)** In each Figure, MFI of the CXCR4 expression without irradiation is defined as a control, and the normalized ratio of each MFI to the control is calculated.

Next, we focused on the effect of carbon-ion irradiation instead of photon irradiation. We detected a minor, insignificant increase in migration with both cell lines following carbon ion doses of 0.3 and 1 Gy (Figure 5A). The relative carbon ion irradiation-associated stimulation of cell migration was significantly inferior to all corresponding photon doses (Figure 5A, */**). To rule out, the observed differences were due to radiation-specific differences in cell survival, we performed clonogenic survival experiments. Both photon and carbon ion irradiation significantly impaired clonogenicity; but there was no significant difference in clonogenic cell survival between both cell lines after irradiation with corresponding photon and carbon doses (Figure 5B). Therefore, cell survival did not account for the result of the radiation modality-specific migratory findings. Moreover, Annexin-V assays showed that no significant difference was found between either cell lines with and without irradiation (data not shown). From these data, loss of clonogenicity or apoptosis were not responsible for the detected migration phenotypes. In addition, FACS analysis revealed that carbon ion irradiation did not stimulate CXCR4 expression on the surface of either cell line as opposed to the photon-induced upregulation of CXCR4 (Figure 5C).

**Figure 6:**
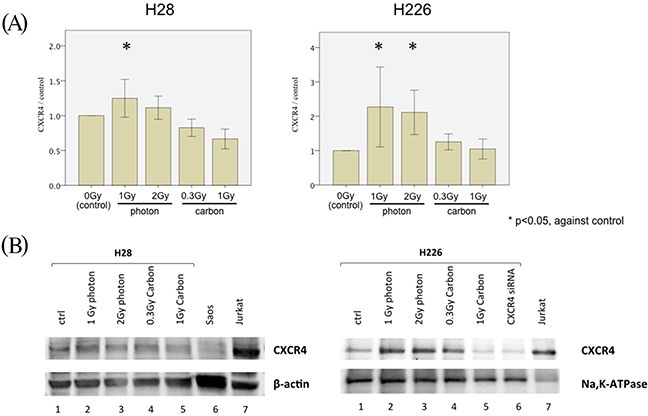
CXCR4 regulation of MPM cell migration following irradiation **(A)** CXCR4 ELISA measuring the concentration of CXCR4 among the total protein lysates generated from H28 cells (left) and H226 cells (right). Cells were treated with a single dose of 1 Gy and 2 Gy photon-irradiation or 0.3 Gy and 1 Gy carbon ion irradiation prior to protein lysate generation. In each Figure, the concentration of CXCR4 is depicted as normalized ratio of measured CXCR4 concentration in analyzed samples to the CXCR4 concentration in protein lysate generated from non-irradiated cells (defined as control). **(B)** Western blot analysis using 20μg of total protein from H28 cells (left) and 20μg membrane-extracted protein from H226 cells (right). H28 and H226 cells were irradiated with a single dose of 1 Gy photon, 2 Gy photon, 0.3 Gy carbon ions and 1 Gy carbon ions before generation of protein lysates. Furthermore, protein was extracted from H226 cells following CXCR4 siRNA treatment. Beta-actin and Na^+^-K^+^ ATPase are used as loading controls of total protein and membrane-extracted protein, respectively. Saos-2 cell lysate are included as negative control and Jurkat cell lysate as positive control for CXCR4 protein expression.

Hypothesizing that the difference in SDF-1α/CXCR4 signaling was responsible for the observed photon vs. carbon ion phenotype, we next performed ELISA, western blots, and knock-down experiments with siRNA to substantiate our findings. CXCR4 ELISA assays confirmed the photon increase of CXCR4 translation in both photon-irradiated cell lines, whereas no such increase was detected in the carbon ion experiments (Figure 6A).

Western blots for CXCR4 in membrane-bound proteins from H226 cells identified the responsible and siRNA-suppressible protein band for CXCR4. Subsequent western blots from both cell lines confirmed an upregulation of CXCR4 translation following photon irradiation with 1 and 2 Gy, while no change in protein levels was detected through western blots following carbon ion doses (Figure 6B).

To address the possible contribution of the CXCR4-ligand SDF-1α, we performed ELISA assays which showed that the concentration of soluble SDF-1α in the supernatants of irradiated cells was below the detection limits in all settings of the cultured cells (data not shown).

## DISCUSSION

We showed that the common clinical finding of marginal MPM recurrences after radiotherapy may be related to photon-induced ECM-based tumor cell hypermigration towards CXCR4-ligands and that this phenomenon may be inhibited either by the addition of CXCR4-antagonists or by replacement of photon irradiation with carbon ion irradiation.

Given the anatomical and pathological charac-teristics in MPM, surgical resection will inevitably result in an incomplete resection of the tumor microscopically [19]. Nevertheless, surgical macroscopic complete resection prolongs survival and, therefore, it plays a significant role in the multimodal therapy of MPM [20]. Photon radiotherapy, commonly administered in the adjuvant setting, is intended to enhance local control rates – but it has repeatedly been reported to cause inacceptable toxicity and to fail in controlling the disease – especially at the borders of prior radiation fields. Speculations about photon irradiation promoting locoregional relapses have previously been supported by findings from several other tumor cell lines.

Signaling between SDF-1α and CXCR4 contributes to tumor growth, angiogenesis, invasion and metastases in numerous malignancies [21–23]. In addition, irradiation-enhanced invasiveness of non-small lung cancer and breast cancer has been attributed to the SDF-1α/CXCR4 interaction [14, 24]. Moreover, the relationship between MPM and the SDF-1α/CXCR4 chemotactic axis has been reported in previous reports [25, 26]. Therefore we selected the CXCR4- SDF-1α pathway as a promising candidate to elucidate the underlying mechanisms of radiation-altered cell migration. To our best knowledge, our study was the first report to demonstrate that photon-induced migration in MPM cells was primarily caused by increased expression of SDF-1α -binding CXCR4.

Since prior findings in irradiated primary brain tumor cells [27, 28], NSCLC cells [29], and non-malignant cells [30] had revealed that increases of integrin cell surface expressions were responsible for radiation-induced hypermigration, we also decided to analyze the expression of various integrins in MPM cells. However, our results showed that no radiation-associated changes of either α_3_-, α_5_-, or β_1_-family integrin expressions, which had previously been reported as collagen I- and IV-binding integrins in MPM [31–33], were responsible for the detected phenotype of photon-increased hypermigration in MPM cells. Therefore, we concluded that the finding of photon-induced MPM cell migration was related to photon-induced increases in responsiveness towards chemoattractants rather than increased MPM cell interactions with the ECM.

Our preclinical data suggest two possible options to overcome this treatment-related adverse event: One option could be the concurrent use of a SDF-1α/CXCR4 inhibitor with photon irradiation. From our results, AMD3100, an FDA approved antagonist of CXCR4, is effective to prevent radiation-promoted migration of MPM cells *in vitro*; therefore, it may be assumed that it may be a promising agent to be investigated in clinical trials that might reduce the rate of marginal/local recurrences in MPM patients following radiotherapy.

A further option may be changing the radiation modality from photon to carbon ion radiotherapy. Carbon ion irradiation offers several advantages when compared with conventional photon radiotherapy, including higher relative biologic effectiveness in tumor cell killing and a more accurate dose distribution, which contributes to the low risk of side effect [34–37], although carbon ion treatment facilities are rare and cause significantly higher treatment costs than conventional photon facilities. Moreover, it has been reported that low doses of carbon ion irradiation attenuate the ability of migration in other malignancies, such as lung cancer [38, 39], pancreatic cancer [40], glioma [27, 41], and fibrosarcoma [18]; therefore, it is believed that carbon ion irradiation may have a different effect on radiation-induced migration compared with conventional photon irradiation. Especially, carbon ion irradiation is known to induce specific subcellular alterations including unique gene expression or enzyme activity profiles. The underlying mechanisms of these alterations have previously been reported, suggesting the activation of the Akt signaling pathway [39] and the inhibition of MMP-2 activity [18, 42]. Our results showed that carbon-ion irradiation did not promote the migration of MPM cell lines, nor did it increase the expression of CXCR4, suggesting that carbon ion irradiation is expected to prevent the recurrence of MPM. For this reason, the use of carbon ion irradiation for MPM patients, especially in an adjuvant lower-dose radiotherapy, should have positive effects and be tested in prospective clinical trials.

Our study has several limitations. First, this study is only an *in vitro* study, which may not accurately reflect the *in vivo* situation. Moreover, the effect of photon irradiation on the microenvironment surrounding the tumor should not be ignored. In order to confirm this mechanism and to address the contribution of microenvironmental modifications through radiation, radiation experiments in animal models with human MPM xenografts would be a promising approach for a subsequent corroborating study [43].

As another limitation, SDF-1α/CXCR4 signaling is not the only pathway to promote tumor cell migration. Other pathways may contribute simultaneously, and the SDF-1α/CXCR4 pathway may work indirectly through further up- and downstream signaling pathways, such as the interaction of other chemokines and chemokine receptors. In this study, we only examined the SDF-1α/CXCR4 pathway and the possible role of integrins, as they have repeatedly been identified to be responsible for radiation-increased cell motility and as there is a pharmacological agent, FDA-approved AMD3100, that can be administered to reverse the observed events.

In order to exclude the potential contribution of further pathways, comprehensive analysis, for example through DNA microarray, RNA-ChiP, or Proteomic analysis, would be required. In the present manuscript, we decided to not perform such experiments, as the detected phenotype was successfully inhibited by adding AMD3100.

In conclusion, low doses of photon irradiation promoted MPM cell migration through the increased expression of CXCR4 with subsequently increased SDF-1α/CXCR4 signaling. Clinically, this might enhance the risk of tumor cell spread and infiltration and, therefore, explain prior disappointing results from clinical trials investigating photon radiotherapy in MPM patients. The administration of the CXCR4 antagonist AMD3100 effectively inhibited this increased migration. As an alternative to photon irradiation, carbon ion irradiation did not significantly promote migration. Therefore, our finding suggest that the co-administration of the clinically already available CXCR4 antagonist AMD3100 concurrently to photon irradiation or, alternatively, the replacement of photon irradiation with carbon ion irradiation may be two possible solutions to establish and enhance the clinical benefit of radiation treatments in MPM patients.

## MATERIALS AND METHODS

### Reagents and cell lines

H28 and H226 mesothelioma cells were purchased from ATCC and maintained at 37°C and 5% CO2 in RPMI 1640 medium supplemented with 1% Penicillin/Streptomycin and 10% fetal bovine serum (FBS; Biochrom, Berlin, Germany). Twenty-four hours before the migration assay, cells were serum starved in RPMI 1640 medium containing 1% Penicillin/Streptomycin and 0.5% FBS. Cell passaging was performed every week.

Stromal cell-derived factor 1α (SDF-1α) was purchased from Gibco (Eggenstein, Germany). The CXC chemokine receptor 4 (CXCR4) antagonist AMD3100 was purchased from Sigma Aldrich (Munich, Germany). To block CXCR4, cells were exposed to AMD3100 at a concentration of 25μg/ml 24 hours before the migration assay.

For FACS analysis, PE-labeled anti-human CXCR4 antibody (555974), PE-labeled anti-human β_1_ antibody (556049), PE-labeled anti-human α_3_ antibody (556025), PE-labeled anti-human α_5_ antibody (555617), and isotope controls corresponding to these antibodies were purchased from BD Bioscience (Heidelberg, Germany). For Western blot analysis, anti- CXCR4 antibody (clone 12G5), and anti- β-actin (clone BA3R) were purchased from Thermo Fisher Scientific (Darmstadt, Germany). Anti- Na+-K+ ATPase (clone EP1845Y) was purchased from Abcam (Cambridge, UK).

### Migration assays using membranes coated with extracellular matrix proteins

For migration assays, polycarbonate membranes with 8-μm pores were coated with 0.5 μg/cm2 Collagen I (Corning, Bodenheim, Germany) and 0.5 μg/cm2 collagen IV (Corning, Bodenheim, Germany) and stored overnight at 4°C before the experiments. Next, 2 × 10^4^ cells were loaded into the upper chamber of a 48-well modified microchemotaxis chamber (Multiwell Chemotaxis Chamber, Neuro Probe). The lower well contained cell culture medium containing 5% FBS, 10% FBS, or SDF-1α (0.1μg/ml), as indicated. An 8-μm pore size polycarbonate membrane separated the lower and upper chambers. After 5 hours of incubation at 37°C, transmigrated cells on the lower chamber side were stained with methylene blue and counted with a Leica DC300F microscope. The number of invading cells was counted using a phase-contrast microscope. Two fields were randomly selected per well, and the number of the cells was recorded by an investigator blinded to experimental set-up.

### Irradiation

Photon irradiation was performed by a biological cabinet X-ray irradiator with 320 kV and 12.50 mA (X-RAD 320 Precision X-ray Inc., N. Bradford, Conn.) at single doses of 1 and 2 Gy (110 cGy/min dose rate).

Carbon-ion irradiation was performed at the Heidelberg Ion Therapy Center using the raster scanning technique developed by Haberer et al. [44] at the horizontal beam line (Siemens AG). An extended Bragg peak (dose average LET, 103 keV/μm) was adjusted using a 30-mm acrylic shield and discharged single doses of 0.3 and 1 Gy. Cell monolayers were positioned in the middle of the extended Bragg peak.

Irradiation was performed at room temperature 24 hours before the experiments were started, as we previously reported [17]. For functional assessment of irradiated cells, cell viability was confirmed using trypan blue staining, and only viable cells were used for migration assay.

Assuming a relative biological effectiveness between 2 and 3, we chose single doses of 0.3 and 1 Gy for carbon-ion irradiations to realize the same biological effect as photon irradiation performed with single doses of 1 and 2 Gy.

### FACS analysis

Twenty-four hours after irradiation, cells were fixed in 4% paraformaldehyde (PFA) in PBS and preserved at 4°C. The fixative was removed by two washes with PBS immediately before staining. Cells were stained with a PE-labeled antibody against β_1_, α_3_, and α_5_ integrins; CXCR4; and matching isotope controls. H28 and H226 mesothelioma cells were analyzed with a flow cytometer (FACS Canto™ II, BD Biosciences, Heidelberg, Germany) and FlowJo software (TreeStar, Ashland, OR, USA). The results are displayed in histogram plots and assessed via subsequent quantitative analyses for the mean fluorescence intensity (MFI).

### Apoptosis assay

Apoptosis of H28/H226 was determined by an Annexin V-PE apoptosis detection kit (BD Biosciences). This assay was performed 24 hours after photon or carbon irradiation, according to the manufacturer's protocol.

### Clonogenic survival assays

Cells were plated and allowed to attach for 24 hours before irradiation. After treatment, cells were grown for 14 days to allow for colony formation. Colonies were fixed with 25% acetic acid in methanol and stained with 0.5% crystal violet solution. Colonies consisting of more than 50 cells were then counted on a light microscope. All clonogenic assays were performed in triplicates.

### ELISA

Concentration of CXCR4 in total cell lysate generated from H28 and H226 cell lines were determined by corresponding ELISA assays (R&D Systems, Wiesbaden, Germany), according to the manufacturers’ protocols.

### Western blot analysis

Cells were grown to 80% confluency in T-75 flask and then treated with the respective irradiation. Total protein and plasma membrane fraction from cells were isolated using Total Protein Extraction Kit and Minute Plasma Membrane Protein Isolation Kit (Invent Biotechnologies, Eden Prairie, MN), respectively. Protein content of the supernatant was determined by the Bradford method with a commercial protein assay reagent (Bio-Rad, Munich, Germany). Sodium dodecyl sulfate-polyacrylamide gel electrophoresis was performed with gels containing 10% polyacrylamide. Proteins were transferred to nitro cellulose membranes. Nitro cellulose membrane was incubated for 1h with 5% BSA in Tris-buffered saline (TBS) and probed with the CXCR4 antibody and the anti- Na+-K+ ATPase over night at 4°C in Tris-buffered saline (TBS) containing 0.1% Tween 20 and 5% BSA. All antibodies were diluted as the recommendation of manufacturer. Membranes were subsequently incubated with secondary antibody conjugated to horseradish peroxidase for 1 hour at room temperature, and immunocomplexes were visualized by enhanced chemiluminescence (Thermo Fisher Scientific). Saos-2 cell lysate (sc-2235) and Jurkat cell lysate (sc-24788), both purchased from Santa Cruz Biotechnology (Heidelberg, Germany), were included in Western blot analysis as negative and positive control for CXCR4 protein expression, respectively.

### RNA silencing of CXCR4

CXCR4 silencing small inhibitory RNA oligon-eucleotides (Thermo Fisher Scientific) were administered according to the manufacturer's protocol. CXCR4 silen-cing small inhibitory RNA represents a mixture of two sequences, 5′-GGCAGUCCAUGUCAUCUAC-3′, and 5′-GUAGAUGACAUGGACUGCC- 3′. Fifty picomol per milliliter of each inhibitory siRNA was administered 24 hours before irradiation.

### Statistical analysis

All experiments were performed at least three times in triplicate in each individual experimental set-up. Comparisons between two groups were performed with Student's t-test (SPSS 23.0; SPSS Inc., Chicago, IL, USA). p-Values less than 0.05 were considered to be statistically significant.
